# Supplementary material for: FCMI-YOLO: An efficient deep learning-based algorithm for real-time fire detection on edge devices
Source: PLoS One. 2025 Aug 7;20(8):e0329555. doi: 10.1371/journal.pone.0329555 (PMC12331093; doi:10.1371/journal.pone.0329555)
Supplement: Attachment — Submitted filename: Response to Reviewers.pdf [file pone.0329555.s001.pdf]

Response to Reviewers' Comments

Manuscript ID: PONE-D-25-01361

Title: FCMI-YOLO: A Deep Learning-Based Algorithm for Real-Time Fire Detection on Edge Devices

Authors: Junjie Lu, Yuchen Zheng, Liwei Guan, Bing Lin, Wenzao Shi, Junyan Zhang and Yunping Wu

We would like to thank the editor for handling the review process of our manuscript. We are very grateful to the reviewers for their thorough review and constructive comments. We have carefully addressed all the review comments and improved the quality of this paper accordingly. Please find below our response to review comments one by one. In the revised manuscript, the sections marked in yellow indicate the changes made.

Response to Reviewer 1's Comments

Comment 1: *The authors should emphasize the merits of the developed method.*

Response: We appreciate the reviewer's suggestion. To emphasize the advantages of the proposed method in this paper, we have taken the following measures:

First, we have emphasized the merits of the proposed FCMI-YOLO algorithm more clearly and thoroughly. Specifically, the following enhancements and advantages have been highlighted:

Algorithmic Improvements:

We designed the FasterNext backbone module, which replaces the original C3 module. This lightweight structure, composed of PConv and PWConv, significantly reduces parameters and computational cost while enhancing feature extraction capabilities in complex environments.

We integrated the Cross-Scale Feature Fusion Module (CCFM) and the Mixed Local Channel Attention (MLCA) mechanism into the neck network. These modules enhance the detection of small fire targets by improving the fusion of hierarchical features and enabling the model to focus on spatially and channel-wise important information.

We introduced the Inner-DIoU loss function, which enhances bounding box regression by adaptively adjusting auxiliary box scales, improving the model's sensitivity to fire target size variations and irregular shapes.

Effectiveness of the Improvements:

As shown in the Ablation Experiments, our improvements led to a 1.5% increase in mAP@0.5, a 5.7% improvement in recall, and a 40% reduction in parameters compared to the YOLOv5s baseline. Furthermore, FLOPs were reduced by 29%, and the algorithm maintained high-speed inference at 91.2 FPS on PC and 23.4 FPS on the Orange Pi 5 Plus edge device.

These results demonstrate that FCMI-YOLO effectively balances accuracy and computational efficiency, making it highly suitable for real-time fire detection in

resource-constrained edge environments.

Furthermore, to comprehensively validate the overall performance of the proposed algorithm, we have systematically expanded Section 4.3.7, Comparison Experiments with Mainstream Algorithms. As shown in Table 1, six additional comparative experiments have been introduced, covering different application scenarios and evaluation dimensions. A detailed comparative analysis has been conducted based on multiple technical metrics, including Precision, FLOPs, and Recall. The experimental results demonstrate that, compared to existing mainstream algorithms, the proposed algorithm exhibits significant advantages in key performance indicators. These quantitative comparisons fully validate the breakthrough progress of our algorithm in achieving a balance between performance and efficiency, providing strong evidence of its superiority. To intuitively verify the effectiveness of FCMI-YOLO, a visual comparison analysis was conducted using the four optimal models from Table 1. As shown in Fig 1, under challenging conditions such as strong light, occlusion ,and long-distance scenarios, YOLOv6, YOLOv9, and YOLOv11 exhibit varying degrees of missed detections. In contrast, FCMI-YOLO consistently achieves the highest detection accuracy across different test scenarios.

Table 1. Performance comparison of mainstream algorithms

Model	Parameters(M)	FLOPs(G)	Recall(%)	mAP@0.5(%)	FPS
Faster R-CNN	136.7	369.7	76.5	67.8	13.5
SSD	23.6	174.8	69.7	86.1	42.3
PP-YOLOEs	7.9	17.36	60.6	88.2	86.4
YOLOv3	62.4	65.9	78.1	82.0	28.9
YOLOv4	64.4	60.6	81.4	87.9	24.3
YOLOv5s	7.0	15.9	78.7	86.5	98.8
YOLOv6s	17.3	44.1	83.3	88.5	77.8
YOLOv7	37.2	105.1	71.3	77.6	78.3
YOLOv7-Tiny	6.0	13.0	83.9	86.4	66.1
YOLOv8s	11.1	28.6	80.7	87.7	84.0
YOLOv8s-World	13.4	32.6	82.2	89.4	36.3
YOLOv8s-FasterNet	8.6	21.8	79.6	86.1	78.4
YOLOv9s	9.9	40.6	82.9	89.0	44.2
YOLOv10s	8.1	24.8	76.7	83.1	48.1
YOLOv11s	9.4	21.5	80.3	88.3	42.5
YOLOv11s-MobileNetv4	5.2	10.5	76.3	84.1	76.0
YOLOv11s-EMO	8.5	11.5	82.0	87.9	31.5
FCMI-YOLO	4.2	11.3	84.4	88.0	91.2

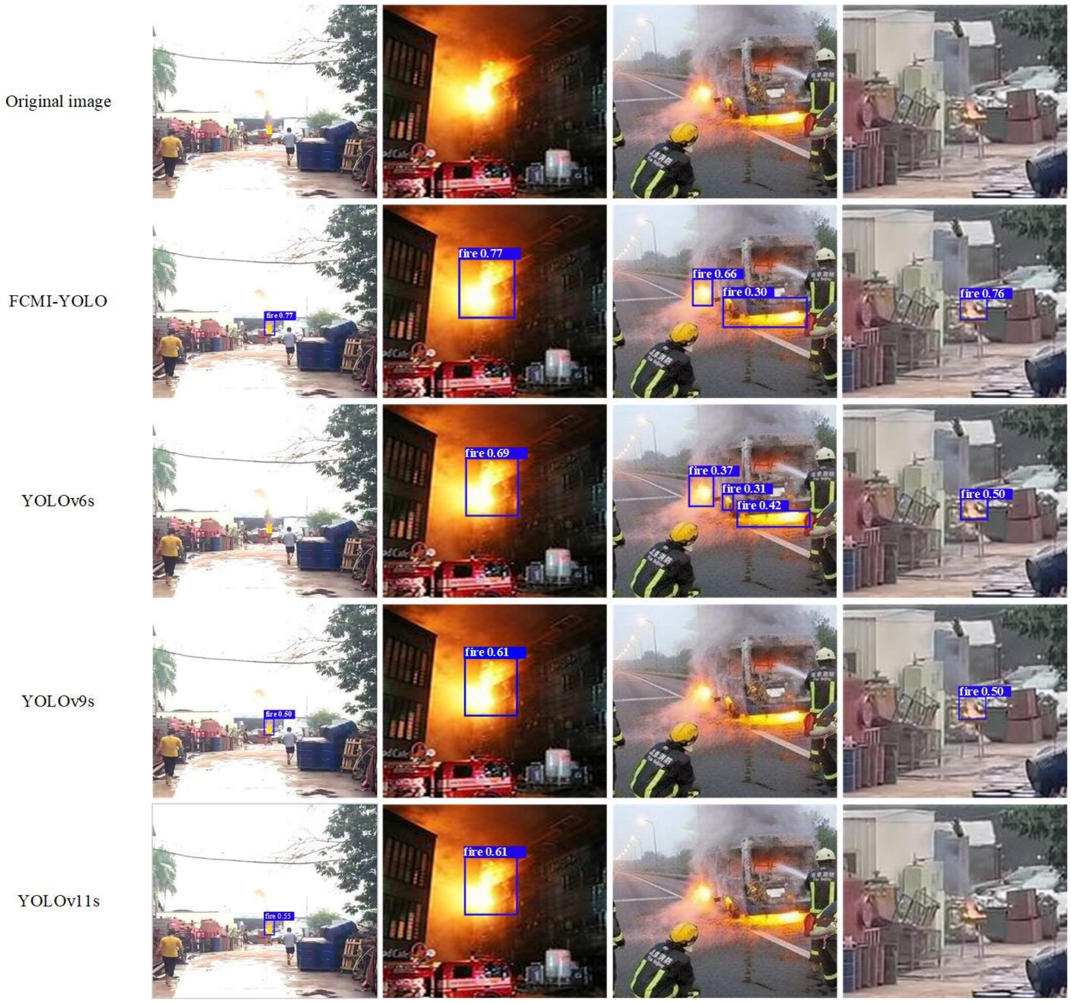

Fig 1. Detection results of FCMI-YOLO, YOLOv6s, YOLOv9s, and YOLOv11s.

[1] Cao X, Su Y, Geng X, Wang Y. YOLO-SF: YOLO for Fire Segmentation Detection. Ieee Access. 2023;11:111079-111092.

[2] Wang S, Wu M, Wei X, Song X, Wang Q, Jiang Y, et al. An advanced multi-source data fusion method utilizing deep learning techniques for fire detection. Engineering Applications of Artificial Intelligence. 2025;142.

[3] He H, Zhang Z, Jia Q, Huang L, Cheng Y, Chen B. Wildfire detection for transmission line based on improved lightweight YOLO. Energy Reports. 2023;9:512-20.

Comment 2: *In the section simulation results, the proposed approach should compare with other relevant algorithms, the authors are asked to further provide comparisons to better show the advantages of their approach.*

Response: We appreciate the reviewer's suggestion. To systematically evaluate the overall performance of the proposed algorithm, we have significantly expanded Section 4.3.7, Comparison Experiments with Mainstream Algorithms. This expansion incorporates six representative advanced object detection algorithms as benchmark references, including PP-YOLOEs [1], YOLOv7-Tiny [2], YOLOv8s-World [3], YOLOv8s-FasterNet [4], YOLOv11s-MobileNetv4 [5], and YOLOv11s-EMO [6], as shown in Table 1.

Table 1. Performance comparison of mainstream algorithms

Model	Parameters(M)	FLOPs(G)	Recall(%)	mAP@0.5(%)	FPS
Faster R-CNN	136.7	369.7	76.5	67.8	13.5
SSD	23.6	174.8	69.7	86.1	42.3
PP-YOLOEs	7.9	17.36	60.6	88.2	86.4
YOLOv3	62.4	65.9	78.1	82.0	28.9
YOLOv4	64.4	60.6	81.4	87.9	24.3
YOLOv5s	7.0	15.9	78.7	86.5	98.8
YOLOv6s	17.3	44.1	83.3	88.5	77.8
YOLOv7	37.2	105.1	71.3	77.6	78.3
YOLOv7-Tiny	6.0	13.0	83.9	86.4	66.1
YOLOv8s	11.1	28.6	80.7	87.7	84.0
YOLOv8s-World	13.4	32.6	82.2	89.4	36.3
YOLOv8s-FasterNet	8.6	21.8	79.6	86.1	78.4
YOLOv9s	9.9	40.6	82.9	89.0	44.2
YOLOv10s	8.1	24.8	76.7	83.1	48.1
YOLOv11s	9.4	21.5	80.3	88.3	42.5
YOLOv11s-MobileNetv4	5.2	10.5	76.3	84.1	76.0
YOLOv11s-EMO	8.5	11.5	82.0	87.9	31.5
FCMI-YOLO	4.2	11.3	84.4	88.0	91.2

To further highlight the advantages of the proposed method, we have added visualization experiments comparing four of the most competitive advanced object detection algorithms (YOLOv6, YOLOv9, YOLOv11, and FCMI-YOLO), as shown in Figure 2. These visual comparison results provide a more comprehensive demonstration of the innovative contributions and technical breakthroughs of this study.

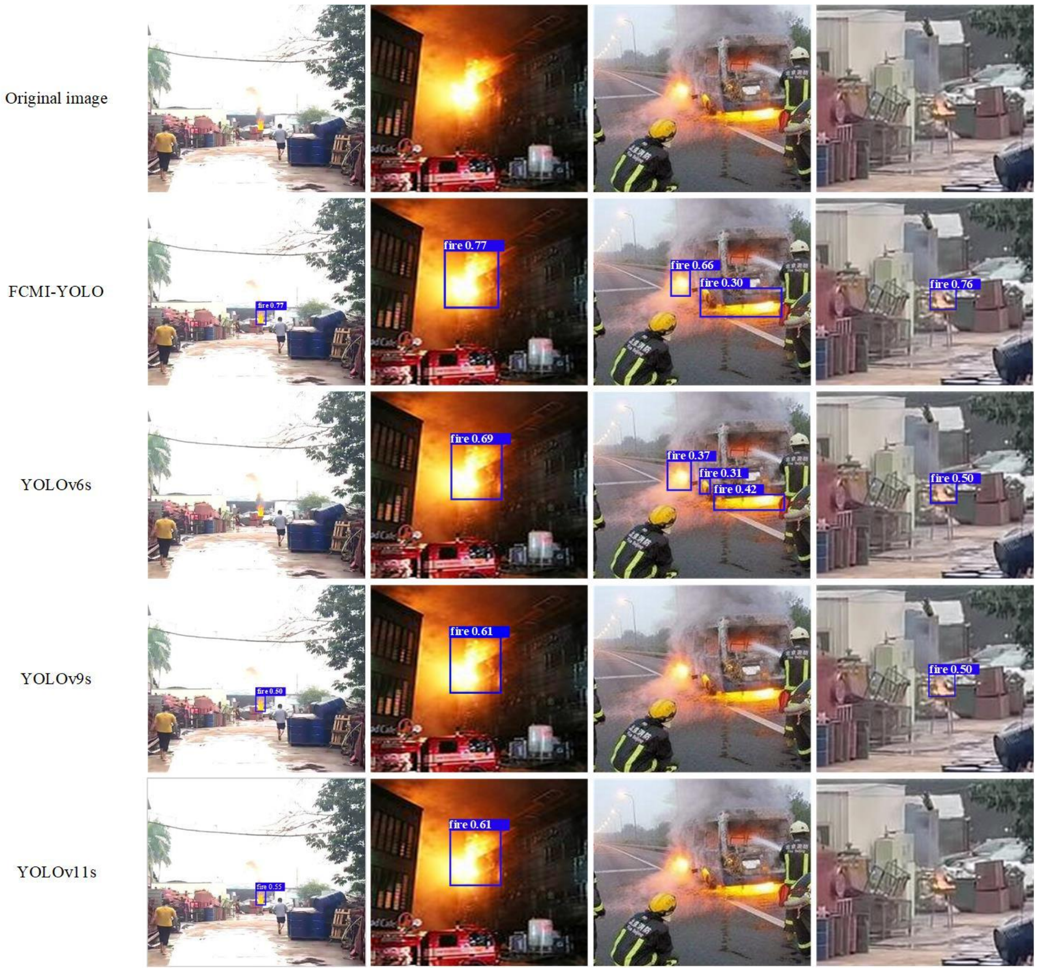

Fig 2. Detection results of FCMI-YOLO, YOLOv6s, YOLOv9s, and YOLOv11s.

[1] Xu S, Wang X, Lv W, Chang Q, Cui C, Deng K, et al. PP-YOLOE: An evolved version of YOLO. arXiv preprint arXiv:2203.16250. 2022.

[2] Zhu J, Zhang J, Wang Y, Ge Y, Zhang Z, Zhang S. Fire Detection in Ship Engine Rooms Based on Deep Learning. Sensors. 2023;23(14).

[3] Cheng T, Sone L, Ge Y, Liu W, Wang X, Shan Y, et al. YOLO-World: Real-Time OpenVocabulary Object Detection. IEEE/CVF Conference on Computer Vision and Pattern Recognition (CVPR); 2024.

[4] Chen J, Kao Sh, He H, Zhuo W, Wen S, Lee CH, et al. Run, don't walk: chasing higher FLOPS for faster neural networks. Proceedings of the IEEE/CVF confer- ence on computer vision and pattern recognition; 2023.

[5] Qin D, Leichner C, Delakis M, Fornoni M, Luo S, Yang F, et al. MobileNetV4: Universal Models for the Mobile Ecosystem. arXiv preprint arXiv:2404.10518. 2024.

[6] Zhang J, Li X, Li J, Liu L, Xue Z, Zhang B, et al. Rethinking Mobile Block for Efficient Attention-based Models. arXiv preprint arXiv:2301.01146. 2023.

Comment 3: *The literature review is not good enough.*

Response: Thank you for your valuable feedback. We have carefully reviewed and revised the Related Work section to ensure that it provides a more comprehensive and detailed literature review. The citation distribution of the related works before and after the revision is illustrated in Figure 1, where the references highlighted in red indicate the newly added or revised citations, while those marked in blue represent the original references that have been retained. Specifically, we have expanded the discussion on existing fire detection algorithms and highlighted recent advancements in YOLO-based methods, as well as other relevant approaches. We have also emphasized the strengths and weaknesses of the methods to clearly establish the research gap that FCMI-YOLO aims to address.

In addition to the existing references, we have included studies that explore the optimization of YOLO models for fire detection in various environments, such as small fire targets, complex scenarios, and real-time deployment on edge devices. These references help contextualize the contributions of our work and demonstrate the advancements made in the field. Moreover, we have discussed the trade-off between accuracy and inference speed, as well as the challenges posed by computational complexity in real-time applications on resource-constrained devices.

To further strengthen the literature review, we have elaborated on the limitations of existing methods and how FCMI-YOLO improves upon them. Specifically, we have discussed how our method effectively balances accuracy and speed while minimizing the computational burden, making it particularly suitable for deployment on edge devices.

We believe these revisions provide a more complete and critical review of the relevant literature and better highlight the necessity and novelty of our proposed approach. Please refer to the updated Related Work section for further details.

Later

13. He H, Zhang Z, Jia Q, Huang L, Cheng Y, Chen B. Wildfire detection for transmission line based on improved lightweight YOLO. Energy Reports. 2023;9:512-20. https://doi.org/10.1016/j.egyr.2022.10.435

14. Chang H-C, Hsu Y-L, Hsiao C-Y, Chen Y-F. Design and Implementation of an Intelligent Autonomous Surveillance System for Indoor Environments. IEEE Sensors Journal. 2021; 21(15):17335-17349. https://doi.org/10.1109/jsen.2021.30 81831

15. Mueller M, Karasev P, Kolesov I, Tannenbaum A. Optical Flow Estimation for Flame Detection in Videos. IEEE Transactions on Image Processing. 2013;22 (7): 2786-2797. https://doi.org/10.3850/978-981-09-5346-1_cse-535

16. Ghassempour N, Zou JJ, He Y. A SIFT-Based Forest Fire Detection Framework Using Static Images. 12th International Conference on Signal Processing and Co- mmunication Systems; 2018.https://doi.org/10.1109/icspcs.2018.8631711

17. Yan M, Yan Y. Fire Detection Based on Improved HOG. 17th Inter- national Symposium on Distributed Computing and Applications for Business Engineering and Science; 2018. https://doi.org/10.1109/dcabes.2018.00038

18. Zhou Z, Wu R. Stock Price Prediction Model Based on Convolutional Neural Networks. Journal of Industrial Engineering and Applied Science. 2024;2(4):1-7. 2018. https://doi.org/10.5281/zenodo.12780145

19. Wu R, Zhang T, Xu F. Cross-Market Arbitrage Strategies Based on Deep Learning. Academic Journal of Sociology and Management. 2024;2(4):20-6. 2018. https://doi.org/10.5281/zenodo.12747401

20. Muhammad K, Ullah A, Lloret J, Del Ser J, De Albuquerque VHC. Deep learning for safe autonomous driving: Current challenges and future directions. IEEE Transactions on Intelligent Transportation Systems. 2020;22(7):4316-36. https://doi.org/10.1109/tits.2020.3032227

21. Muruganantham P, Wibowo S, Grandhi S, Samrat NH, Islam N. A systematic literature review on crop yield prediction with deep learning and remote sensing. Remote Sensing. 2022;14(9):1990. https://doi.org/10.3390/rs14091990

22. Duong H-T, Le V-T, Hoang VT. Deep learning-based anomaly detection in video surveillance: A survey. Sensors. 2023;23(11):5024. https://doi.org/10.3390/s23115024

23. Younan M, Houssein EH, Elhoseny M, Ali AA. Challenges and recommended technologies for the industrial internet of things: A comprehensive review. Measurement. 2020; 151:107198. https://doi.org/10.1016/j.measurement.2019.107198

24. Alakbari FS, Mohyaldinn ME, Ayoub MA, Muhsan AS. Deep learning approach for robust prediction of reservoir bubble point pressure. ACS omega. 2021;6(33):21499-513. https://doi.org/10.1021/acsomega.1c02376.s001

25. Alakbari FS, Mohyaldinn ME, Ayoub MA, Muhsan AS, Abdulkadir SJ, Hussein IA, et al. Prediction of critical total drawdown in sand production from gas wells: Machine learning approach. The Canadian Journal of Chemical Engineering. 2023;101(5):2493-509. https://doi.org/10.1002/cjce.24640

26. Alakbari FS, Mohyaldinn ME, Ayoub MA, Hussein IA, Muhsan AS, Ridha S, et al. A gated recurrent unit model to predict Poisson's ratio using deep learning. Journal of Rock Mechanics and Geotechnical Engineering. 2024;16(1):123-35. https://doi.org/10.1016/j.jrmge.2023.04.012

27. Ren S, He K, Girshick R, Sun J. Faster R-CNN: Towards real-time object detection with region proposal networks. . IEEE transactions on pattern analysis and machine intelligence. 2016;39(6):1137-1149.https://doi.org/10.1109/TPAMI.2016. 2577031

28. Girshick R. Fast r-cnn. arXiv preprint arXiv:150408083. 2015. https://doi.org/10.1109/ICCV.2015.169

29. Redmon J. You only look once: Unified, real-time object detection. Proceedings of the IEEE conference on computer vision and pattern recognition;2016. https://doi.org/10.1109/CVPR.2016.91

30. Liu W, Anguelov D, Erhan D, Szegedy C, Reed S, Fu C-Y, et al. Ssd: Single shot multibox detector. 14th European Conference on Computer Vision; 2016.

31. Johnston J, Zeng K, Wu N. An evaluation and embedded hardware implement- ation of yolo for real-time wildfire detection. 2022 IEEE 12th World AI IoT Congress; 2022. https://doi.org/10.1109/aiiot54504.2022.9817206

32. Zheng H, Dembele S, Wu Y, Liu Y, Chen H, Zhang Q. A lightweight algorithm capable of accurately identifying forest fires from UAV remote sensing imagery. Frontiers in Forests and Global Change. 2023;6:1134942. https://doi.org/10.338 9/ffgc.2023.1134942

33. Li Y, Shang J, Yan M, Ding B, Zhong J. Real-time early indoor fire detection and localization on embedded platforms with fully convolutional one-stage object detection. Sustainability. 2023;15(3):1794.https://doi.org/10.3390/su15031794

34. Chan Y, Liu J, Kristiani E, Lien K, Yang C. Flame and smoke detection using Kafka on edge devices. Internet of Things. 2024;27:101309. https://doi.org/10.1016/j.iot.204.101309

35. Jiang X, Xu L, Fang X. DG-YOLO: A Novel Efficient Early Fire Detection Algorithm Under Complex Scenarios. Fire Technology. 2024. https://doi.org/10.1007/s10694-024-01672-z

36. Xu R, Lin H, Lu K, Cao L, Liu Y. A Forest Fire Detection System Based on Ensemble Learning. Forests. 2021;12(2). https://doi.org/10.3390/f12020217

37. Luan T, Zhou S, Zhang G, Song Z, Wu J, Pan W. Enhanced Lightweight YOLOX for Small Object Wildfire Detection in UAV Imagery. Sensors. 2024;24(9). https://doi.org/10.3390/s24092710

38. Zhao H, Jin J, Liu Y, Guo Y, Shen Y. FSDF: A high-performance fire detection framework. Expert Systems with Applications. 2024;238. https://doi.org/10.1016/j.eswa.2023.121665

39. Wang Y, Hua C, Ding W, Wu R. Real-time detection of flame and smoke using an improved YOLOv4 network. Signal Image and Video Processing. 2022;16(4): 1109- 1116. https://doi.org/10.1007/s11760-021-02060-8

40. Huang L, Ding Z, Zhang C, Ye R, Yan B, Zhou X, et al. YOLO-ULNet: Ultralightweight Network for Real-Time Detection of Forest Fire on Embedded Sensing Devices. Ieee Sensors Journal. 2024;24(15):25175-25185.https://doi.org/10.1109/j sen.2024.3416548

Before

29. Yang J, Zhu W, Sun T, Ren X, Liu F. Lightweight forest smoke and fire detect- ion algorithm based on improved YOLOv5. PLoS one.2023;18(9):e0291359. https://doi.org/10.1371/journal.pone.0291359

30. Wang Z. Enhanced Fire Detection Algorithm for Chemical Plants Using Modified YOLOv9 Architecture. 9th International Conference on Electronic Technology and Information Science; 2024. https://doi.org/10.1109/icetis61828.2024.10593793

31. Akhmedov F, Nasimov R, Abdusalomov A. Dehazing Algorithm Integration with YOLO-v10 for Ship Fire Detection. Fire-Switzerland. 2024;7(9):332.https://doi.org/10.3390/fire7090332

32. Wani JA, Sharma S, Muzamil M, Ahmed S, Sharma S, Singh S. Machine learning and deep learning based computational techniques in automatic agricultural dis- eases detection: Methodologies, applications, and challenges. Archives of Compu- tational methods in Engineering. 2022;29(1):641-677.https://doi.org/10.1007/s11831-021-09588-5

33. Muruganantham P, Wibowo S, Grandhi S, Samrat NH, Islam N. A systematic literature review on crop yield prediction with deep learning and remote sensing. Remote Sensing. 2022;14(9):1990.https://doi.org/10.3390/rs14091990

34. Duong HT, Le VT, Hoang VT. Deep learning-based anomaly detection in video surveillance: A survey. Sensors. 2023;23(11):5024.https://doi.org/10.3390/s23115 024

35. Younan M, Houssein EH, Elhoseny M, Ali AA. Challenges and recommended technologies for the industrial internet of things: A comprehensive review. Measurement. 2020;151:107198.https://doi.org/10.1016/j.measurement.2019.107198

36. Jiang Y, Yang K, Zhu J, Qin L. YOLO-Rlepose: Improved YOLO Based on Swin Transformer and Rle-Oks Loss for Multi-Person Pose Estimation. Electronics. 2024;13(3):563.https://doi.org/10.3390/electronics13030563

37. Zhang Q, Zhang J, Li Y, Zhu C, Wang G. IL-YOLO: An Efficient Detection Algorithm for Insulator Defects in Complex Backgrounds of Transmission Lines. Ieee Access. 2024;12:14532-14546.https://doi.org/10.1109/access.2024.3358205

38. Ren R, Sun H, Zhang S, Zhao H, Wang L, Su M, et al. FPG-YOLO: A detection method for pollenable stamen in 'Yuluxiang' pear under non-structural environm- ents. Scientia Horticulturae. 2024;328:112941.https://doi.org/10.1016/j.scienta.20 24.112941

39. Xu H, Li B, Zhong F. Light-YOLOv5: A lightweight algorithm for improved YOLOv5 in complex fire scenarios. Applied Sciences. 2022;12(23):12312. https://doi.org/10.3390/app122312312

40. Chen Y, Li J, Sun K, Zhang Y. A lightweight early forest fire and smoke detect- ion method. Journal of Supercomputing. 2024;80(7):9870-9893. https://doi.org/10.1007/s11227-023-05835-7

41. Kong D, Li Y, Duan M. Fire and smoke real-time detection algorithm for coal mines based on improved YOLOv8s. Journal of Supercomputing. 2024;80(7):98 70-9893. https://doi.org/10.1371/journal.pone.0300502

42. Xu B, Hua F, Yang Q, Wang T. YOLO-Fire: A Fire Detection Algorithm Based on YOLO. 20th International Conference on Intelligent Computing; 2024.

43. Wang Y, Hua C, Ding W, Wu R. Real-time detection of flame and smoke using an improved YOLOv4 network. Signal Image and Video Processing. 2022;16(4): 1109-1116. https://doi.org/10.1007/s11760-021-02060-8

44. Huang L, Ding Z, Zhang C, Ye R, Yan B, Zhou X, et al. YOLO-ULNet: Ultral- ightweight Network for Real-Time Detection of Forest Fire on Embedded Sensing Devices. Ieee Sensors Journal. 2024;24(15):25175-25185.https://doi.org/10.1109/jsen.2024.3416548

Fig 1. The citation distribution of the related works before and after the revision.

Comment 4: *Some minor grammatical errors should be eliminated .the authors are kindly asked to double check the manuscript.*

Response: We sincerely appreciate your meticulous review of the manuscript's language quality. Our team members have conducted multiple rounds of cross-checking, focusing on correcting grammar, spelling, and sentence structure issues while ensuring consistency in the use of academic terminology. Throughout the revision process, we paid special attention to the accuracy of academic English expressions and the fluency of the writing, striving to meet the professional standards required by the journal. In the revised version, all language-related modifications have been clearly highlighted to facilitate review. Your valuable feedback has significantly contributed to improving the quality of our paper, for which we are deeply grateful. If you identify any further areas for improvement, we will fully cooperate in making the necessary revisions.

Comment 5: *The abstract could be more concise and the point.*

Response: Thank you for your valuable suggestion. In response, we have revised the Abstract to be more concise and focused on the key contributions of our work. The updated version highlights the core innovations of FCMI-YOLO and its performance improvements in a streamlined manner. Below is the revised version:

The rapid development of Internet of Things (IoT) technology and deep learning has propelled the deployment of vision-based fire detection algorithms on edge devices, significantly exacerbating the trade-off between accuracy and inference speed under hardware resource constraints. To address this issue, this paper proposes a real-time fire detection algorithm named FCMI-YOLO. Firstly, the FasterNext model, which is based on FasterNet, is introduced to reduce the computational of model parameters and enhance detection precision through lightweight design. Secondly, the Cross-Scale Feature Fusion Module (CCFM) and the Mixed Local Channel Attention (MLCA) mechanism are incorporated into the neck network, significantly improving detection performance for small fire targets and minimizing resource consumption. Finally, the Inner-DIoU loss function is proposed to optimize bounding box regression by incorporating auxiliary boxes. Experimental results on the fire dataset we constructed demonstrate that FCMI-YOLO increases mAP@50 by 1.5%, decreases the number of parameters by 40%, and lowers GFLOPs to 28.9% of those in YOLOv5s. These results demonstrate that FCMI-YOLO enhances fire detection accuracy while significantly reducing computational demands, making it highly valuable for widespread practical applications, particularly on resource-constrained edge devices.

If you have any further suggestions, we will continue to refine it.

Comment 6: *I suggest the authors consider exposes the equation more clearly so to achieve a broader audience.*

Response: We sincerely appreciate your valuable suggestions. Based on your feedback, we have comprehensively improved the presentation of formulas in our paper. In Section 3.2, FasterNext Network, to more intuitively demonstrate the advantages of the SiLU activation function, we have added a comparative formulation between ReLU and SiLU (as shown in

Equations (1) and (2)). Through a formal mathematical expression, we illustrate their differences in key properties such as gradient continuity and output smoothness. Additionally, we incorporate experimental data to quantitatively analyze the improvements SiLU brings to model convergence speed and detection accuracy.

$$\mathrm{Re}LU(x) = \max(0, x) \tag{1}$$

$$SiLU(x) = \frac{x}{1 + e^{-x}} \tag{2}$$

Additionally, in Section 3.4, Improved Loss Function, we have systematically derived the complete mathematical expression of the CIoU loss function (as shown in Equations (3) – (5)). Furthermore, we have conducted an in-depth analysis of key components such as the aspect ratio term, explaining their physical significance and their impact on the bounding box regression mechanism.

$$IoU = \frac{inter}{union} \tag{3}$$

$$\nu = \frac{4}{\pi^2}\left(\arctan\frac{w^{gt}}{h^{gt}} - \arctan\frac{w}{h}\right)^2 \tag{4}$$

$$\alpha = \frac{\nu}{(1 - IoU) + \nu} \tag{5}$$

By presenting clearer mathematical expressions, we have enhanced the readability of the content, allowing readers from diverse academic backgrounds to better grasp our methodological innovations. Once again, we sincerely appreciate your constructive feedback, which is crucial to improving the quality of our paper. If further adjustments are needed, we will be happy to make the necessary refinements.

Comment 7: *Please add more sentences to describe the limitations of this work. The advantages and disadvantages should be discussed.*

Response: Thank you for your valuable feedback. We have expanded the discussion on the limitations of our research in the Conclusion section of the paper.

Currently, the algorithm’s detection accuracy needs improvement when dealing with complex environments such as distant fire incidents, strong winds, intense light, and reflective surfaces. Although the model has been optimized in terms of parameter size and computational cost, its inference speed still requires further enhancement to meet more stringent real-time application requirements. Additionally, due to hardware performance differences across edge devices, the algorithm's performance varies on different platforms, presenting new challenges for cross-platform optimization. To address these limitations, future research will focus on improving the generalization ability of FCMI-YOLO in diverse detection scenarios, exploring more efficient lightweight strategies to optimize detection speed, and developing deployment solutions that can adapt to different hardware platforms. These additional discussions will help readers more objectively assess the applicability and development potential of this research.

The updated content is as follows:

"First, its detection accuracy may decrease when fire targets are at long distances or in adverse environments, such as strong wind, intense light, or reflective surfaces. Second,

although the model has been optimized for parameters and computational cost, there is still room for further improvement in inference speed for real-time applications. Lastly, the performance on edge devices varies depending on the hardware, making model optimization for different platforms a challenge for future work. Future research will focus on enhancing the generalization ability of FCMI-YOLO to adapt to a wider range of detection scenarios. Additionally, further lightweight strategies will be explored to improve detection speed regarding FPS. "

Response to Reviewer 2's Comments

Comment 1: *The title should be improved.*

Response: Thank you for your suggestion. In response, we have revised the title to better reflect the efficiency aspect of our proposed method. The new title is: "FCMI-YOLO: An Efficient Deep Learning-Based Algorithm for Real-Time Fire Detection on Edge Devices."

This revision emphasizes the efficiency of the FCMI-YOLO algorithm, aligning with the core contributions of the paper.

Comment 2: *The objectives and the rationale of the study are recommended to be clearly stated.*

Response: Thank you for your valuable suggestion. In response, we have revised the manuscript to more clearly present the research objectives and the rationale behind our study, with a particular emphasis on the core challenges currently facing the field.

In the Abstract, we refined the background description to highlight the primary challenge this study addresses, the trade-off between accuracy and inference speed under limited computational resources. Specifically, we state: "The rapid development of Internet of Things (IoT) technology and deep learning has propelled the deployment of vision-based fire detection algorithms on edge devices, significantly exacerbating the trade-off between accuracy and inference speed under hardware resource constraints." This clearly defines the technical bottleneck that motivates our work.

In the Introduction, we strengthened the rationale by analyzing the limitations of several recently published approaches. For example, Cao et al. [1] and Wang et al. [2] enhanced detection accuracy but introduced heavier models, making them unsuitable for real-time edge deployment. On the other hand, He et al. [3] attempted to reduce complexity but at the cost of performance robustness. These comparisons allow us to better frame the challenge: existing fire detection algorithms either sacrifice accuracy for speed or vice versa, and few achieve both on embedded systems. To address this, we propose FCMI-YOLO, a real-time algorithm that strikes a better balance between detection precision and computational efficiency through architectural improvements and loss function optimization.

In the Related Work section, we further elaborated on this challenge by integrating a comprehensive review of YOLO-based fire detection methods. Specifically, we discussed how approaches such as deformable attention (Jiang et al. [4]), ensemble learning (Xu et al. [5]), multi-branch fusion (Luan et al. [6]), and unsupervised dual-path frameworks (Zhao et al. [7]) have achieved performance improvements but often introduced substantial increases in

complexity or hardware requirements. Moreover, although some lightweight models (e.g., Wang et al. [8], Huang et al. [9]) show potential for edge deployment, they still suffer from constrained detection accuracy in complex fire scenes such as long-range, small-scale, or occluded targets.

To summarize, our revisions more explicitly define the central challenge, how to design a fire detection algorithm that is accurate, lightweight, and truly deployable on edge devices. FCMI-YOLO is presented as a targeted solution to this issue. These revisions ensure that the research objectives and rationale are clearly stated and well-supported. Please refer to the Abstract, Introduction, and Related Work sections for further details.

[1] Cao X, Su Y, Geng X, Wang Y. YOLO-SF: YOLO for Fire Segmentation Detection. Ieee Access. 2023;11:111079-111092.

[2] Wang S, Wu M, Wei X, Song X, Wang Q, Jiang Y, et al. An advanced multi-source data fusion method utilizing deep learning techniques for fire detection. Engineering Applications of Artificial Intelligence. 2025;142.

[3] He H, Zhang Z, Jia Q, Huang L, Cheng Y, Chen B. Wildfire detection for transmission line based on improved lightweight YOLO. Energy Reports. 2023;9:512-20.

[4] Jiang X, Xu L, Fang X. DG-YOLO: A Novel Efficient Early Fire Detection Algorithm Under Complex Scenarios. Fire Technology. 2024.

[5] Xu R, Lin H, Lu K, Cao L, Liu Y. A Forest Fire Detection System Based on Ensemble Learning. Forests. 2021;12(2).

[6] Luan T, Zhou S, Zhang G, Song Z, Wu J, Pan W. Enhanced Lightweight YOLOX for Small Object Wildfire Detection in UAV Imagery. Sensors. 2024;24(9).

[7] Zhao H, Jin J, Liu Y, Guo Y, Shen Y. FSDF: A high-performance fire detection framework. Expert Systems with Applications. 2024;238.

[8] Wang Y, Hua C, Ding W, Wu R. Real-time detection of flame and smoke using an improved YOLOv4 network. Signal Image and Video Processing. 2022;16(4): 1109- 1116.

[9] Huang L, Ding Z, Zhang C, Ye R, Yan B, Zhou X, et al. YOLO-ULNet: Ultralightweight Network for Real-Time Detection of Forest Fire on Embedded Sensing Devices. Ieee Sensors Journal. 2024;24(15):25175-25185.

Comment 3: *The concluding remarks of the abstract are not well-written. It's merely the repetition of the objectives and title of the manuscript. Please add method limitations and justification to the abstract.*

Response: We sincerely thank the reviewer for this valuable comment. As suggested, we have revised the concluding part of the Abstract to avoid simple repetition of the objectives and title. Specifically, we have added a brief explanation of the limitations of the proposed method and provided justification for its effectiveness. The following modifications were made in the Abstract:

"The rapid development of Internet of Things (IoT) technology and deep learning has propelled the deployment of vision-based fire detection algorithms on edge devices, significantly exacerbating the trade-off between accuracy and inference speed under hardware resource constraints."

This sentence highlights a core limitation in current approaches and provides context for

our method. Furthermore, we have justified the rationality of the proposed improvements by adding:

"Experimental results on the fire dataset we constructed demonstrate that FCMI-YOLO increases mAP@50 by 1.5%, decreases the number of parameters by 40%, and lowers GFLOPs to 28.9% of those in YOLOv5s. These results demonstrate that FCMI-YOLO enhances fire detection accuracy while significantly reducing computational demands, making it highly valuable for widespread practical applications, particularly on resource-constrained edge devices."

In addition, the limitations of the method are discussed in more detail in the Conclusion section of the revised manuscript. Specifically, we have pointed out the performance decline in challenging environments, limitations in inference speed, and the challenges posed by hardware heterogeneity on edge platforms.

We appreciate the reviewer's insightful suggestion, which helped us to enhance the quality of our manuscript.

Comment 4: *The innovation of using this study is not very clear. I do not see a clear reason that this study can perform better than others. Why did the authors choose the method for this study?*

Response: We appreciate the reviewer's suggestion. In this study, the proposed FCMI-YOLO is designed to achieve an optimal trade-off between detection accuracy and inference speed, particularly for deployment on resource-constrained edge devices.

1.The innovation of our study is embodied in three major enhancements that collectively enable FCMI-YOLO to achieve superior performance for real-time fire detection on edge devices by addressing the trade-off between accuracy and inference speed:

(1) FasterNext Module

The original YOLOv5s uses C3 modules with Bottleneck modules, which suffer from redundant parameters and limited feature extraction in complex fire scenarios. Inspired by FasterNet, we propose FasterNext, replacing C3 with a lightweight architecture combining Partial Convolution (PConv) and Pointwise Convolution (PWConv). PConv reduces FLOPs to 1/16 of standard convolutions by processing only a subset of channels, while SiLU activation enhances nonlinear feature learning.

As shown in Table 1, FasterNext reduces the parameter count by 31% compared to C3. As indicated in Table 2, after incorporating FasterNext, YOLOv5s experiences only a slight reduction in FPS, resulting in a decrease in both parameter count and floating-point operations, while simultaneously improving both accuracy and recall rate.

Table 1. Parameters of the FasterNext and C3.

Model Name	Img Size	Channels	Parameters
C3	640×640	(256,256)	296448
FasterNext	640×640	(256,256)	202240

Table 2. Performance comparison of YOLOv5s with FasterNext module replacement.

Backbone	Neck	P(%)	R(%)	mAP@0.5	mAP@0.5:0.95	Parameters(M)	FLOPs(M)	FPS
C3	C3	87.2	78.7	86.5	47.8	7.0	15946	98.8
FasterNext	C3	89.1	83.4	87.8	48.4	6.3	13836	93.3
C3	FasterNext	89.1	84.5	87.2	46.5	6.4	14740	98.2
FasterNext	FasterNext	88.5	83.7	86.9	46.4	5.7	12630	88.9

(2) Cross-Scale Feature Fusion Module (CCFM) and Mixed Local Channel Attention (MLCA)

The Path Aggregation Network (PAN) structure in YOLOv5 struggles with detecting small fire targets and suffers from computational inefficiency. To address these issues, the Cross-Scale Feature Fusion Module (CCFM) employs lightweight convolutions for multi-scale feature fusion, preserving spatial details crucial for small target detection. Additionally, the Mixed Local Channel Attention (MLCA) integrates spatial and channel attention through 1x1 convolutions, focusing on critical fire regions while maintaining low computational overhead.

As shown in Table 3, introducing the CCFM and MLCA mechanisms in the YOLOv5s neck network resulted in a 4.7% improvement in recall and a noticeable reduction in false positives, especially in scenarios with small fire targets or complex backgrounds, where the detection performance became more stable. This improvement is largely due to CCFM enhancing the interaction between high and low-level features through cascaded feature fusion, which improves the representation of small fire targets. Meanwhile, MLCA, by combining spatial and channel information, allows the network to focus more effectively on key fire features while suppressing background noise. Furthermore, this optimization reduced the model parameters by 30% and FLOPs by 15.9%, demonstrating higher computational efficiency that meets the stringent resource constraints of edge devices.

Table 3. Ablation experiments results of YOLOv5s.

FasterNext	CCFM	MLCA	Inner-DIoU	P	R	mAP@0.5	Parameters	FLOPs(M)	FPS
				87.2	78.7	86.5	7.0	15946	98.8
✓				89.1	83.4	87.8	6.3	13836	93.3
	✓			88.7	82.3	86.9	4.9	13390	96.1
		✓		86.7	83.4	87.1	7.0	15984	98.0
			✓	89.2	81.9	87.4	7.0	15946	99.2
	✓	✓		87.2	83.4	86.6	4.9	13428	95.3
✓	✓			86.7	81.9	85.9	4.2	11280	96.0
✓	✓	✓		88.2	82.4	87.8	4.2	11299	90.8
✓	✓	✓	✓	88.4	84.4	88.0	4.2	11299	91.2

(3) Inner-DIoU Loss Function

In the YOLOv5s algorithm, the output layer utilizes CIoU to compute the bounding box loss. However, CIoU lacks sensitivity to scale variations in irregular fire shapes. To address this limitation, Inner-DIoU introduces auxiliary bounding boxes scaled by a dynamic ratio parameter, enhancing regression for both high- and low-IoU samples. By incorporating a scale-adjusting factor, Inner-DIoU dynamically modifies the aspect ratio of the auxiliary bounding box to optimize detection performance.

As shown in Table 4, Inner-DIoU achieves the highest mAP@0.5 (88.0%) and recall (84.4%) among the evaluated loss functions. As indicated in Table 5, Inner-DIoU effectively enhances fire recognition accuracy, reduces false detections, and improves the model's sensitivity to scale variations. Specifically, it increases precision, recall, and mAP@0.5 by 2%, 3.2%, and 0.9%, respectively. This improvement is attributed to the dynamic adjustment of scaling parameters, which enhances the network's adaptability to varying fire target sizes, with particularly significant recall gains in scenarios involving large-scale variations.

By redefining the bounding box regression mechanism, Inner-DIoU strengthens the

model's ability to capture multi-scale fire features, improving target differentiation while mitigating the localization bias caused by scale sensitivity in traditional methods. This results in a synergistic optimization of both model robustness and detection accuracy.

Table 4. Performance comparison of different loss functions.

Loss Function	mAP@0.5 (%)	R (%)
CIoU	87.3	82.4
DIoU	87.3	82.9
EIoU	86.8	81.4
GIoU	87.5	81.1
Focal-CIoU	86.3	85.0
Focal-DIoU	87.5	83.9
Focal-EIoU	87.0	84.0
Focal-GIoU	87.0	83.3
Inner-CIoU	86.6	82.2
Inner-DIoU	88.0	84.4
Inner-EIoU	85.2	78.8
Inner-GIoU	87.1	83.7

Table 5. Ablation experiments results of YOLOv5s.

FasterNext	CCFM	MLCA	Inner-DIoU	P	R	mAP@0.5	Parameters	FLOPs(M)	FPS
				87.2	78.7	86.5	7.0	15946	98.8
✓				89.1	83.4	87.8	6.3	13836	93.3
	✓			88.7	82.3	86.9	4.9	13390	96.1
		✓		86.7	83.4	87.1	7.0	15984	98.0
			✓	89.2	81.9	87.4	7.0	15946	99.2
	✓	✓		87.2	83.4	86.6	4.9	13428	95.3
✓	✓			86.7	81.9	85.9	4.2	11280	96.0
✓	✓	✓		88.2	82.4	87.8	4.2	11299	90.8
✓	✓	✓	✓	88.4	84.4	88.0	4.2	11299	91.2

2. Advantages of FCMI-YOLO Over Existing Methods

In Section 4.3.7, Comparison of Mainstream Algorithms, FCMI-YOLO is evaluated against mainstream algorithms, including Faster R-CNN [1], SSD [2], PP-YOLOEs [3], YOLOv3 [4], YOLOv4 [5], YOLOv5s [6], YOLOv6s [7], YOLOv7 [8], YOLOv7-Tiny[9], YOLOv8s [10], YOLOv8s-World [11], YOLOv8s-FasterNet [12], YOLOv9s [13], YOLOv10s [14], YOLOv11s [15], YOLOv11s-MoibleNetV4 [16], YOLOv11s-EMO[17].

As shown in Table 9, FCMI-YOLO outperforms these models by achieving an optimal trade-off between accuracy, inference speed, and computational efficiency, making it particularly well-suited for real-time fire detection on edge devices. It attains the highest recall (84.4%), ensuring reliable detection in complex environments, which is a crucial factor for early fire warning systems. While its precision is slightly lower than a few high-precision models, FCMI-YOLO significantly reduces model parameters and flops, enabling fast inference at 91.2 FPS while maintaining competitive accuracy (88.0% mAP@0.5). Compared to mainstream algorithms, it demonstrates superior performance with a lightweight architecture, offering strong adaptability for embedded deployment without compromising detection effectiveness.

To intuitively verify the effectiveness of FCMI-YOLO, a visual comparison analysis was conducted using the four optimal models from Table 6. As shown in Fig 13, under challenging conditions such as strong light, occlusion, and long-distance scenarios, YOLOv6, YOLOv9, and YOLOv11 exhibit varying degrees of missed detections. In contrast, FCMI-YOLO consistently achieves the highest detection accuracy across different test scenarios.

Table 6. Performance comparison of mainstream algorithms.

Model	Parameters(M)	FLOPs(G)	Recall(%)	mAP@0.5(%)	FPS
Faster R-CNN	136.7	369.7	76.5	67.8	13.5
SSD	23.6	174.8	69.7	86.1	42.3
PP-YOLOEs	7.9	17.36	60.6	88.2	86.4
YOLOv3	62.4	65.9	78.1	82.0	28.9
YOLOv4	64.4	60.6	81.4	87.9	24.3
YOLOv5s	7.0	15.9	78.7	86.5	98.8
YOLOv6s	17.3	44.1	83.3	88.5	77.8
YOLOv7	37.2	105.1	71.3	77.6	78.3
YOLOv7-Tiny	6.0	13.0	83.9	86.4	66.1
YOLOv8s	11.1	28.6	80.7	87.7	84.0
YOLOv8s-World	13.4	32.6	82.2	89.4	36.3
YOLOv8s-FasterNet	8.6	21.8	79.6	86.1	78.4
YOLOv9s	9.9	40.6	82.9	89.0	44.2
YOLOv10s	8.1	24.8	76.7	83.1	48.1
YOLOv11s	9.4	21.5	80.3	88.3	42.5
YOLOv11s-MobileNetv4	5.2	10.5	76.3	84.1	76.0
YOLOv11s-EMO	8.5	11.5	82.0	87.9	31.5
FCMI-YOLO	4.2	11.3	84.4	88.0	91.2

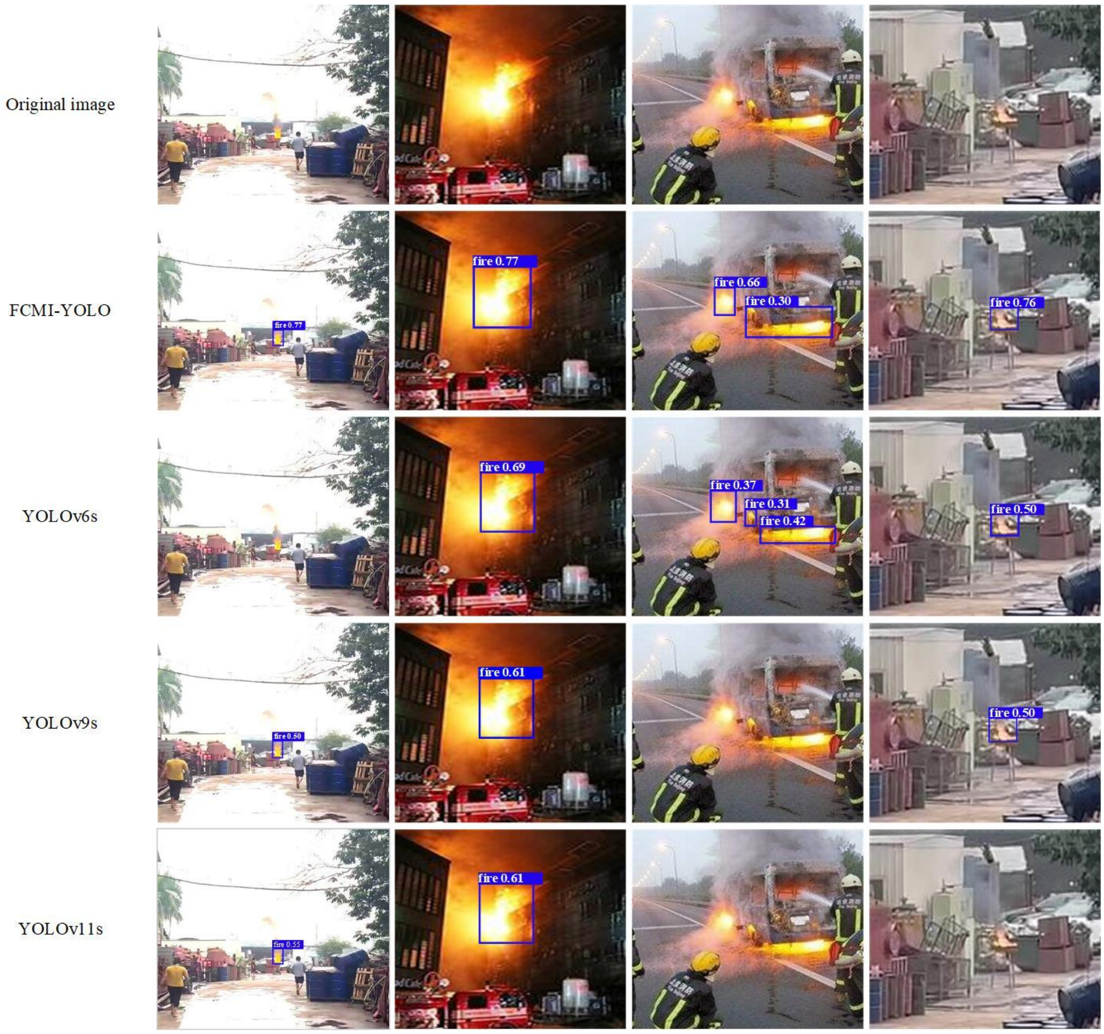

Fig 1. Detection results of FCMI-YOLO, YOLOv6s, YOLOv9s, and YOLOv11s.

[1] Ren S, He K, Girshick R, Sun J. Faster R-CNN: Towards real-time object detection with region proposal networks. IEEE transactions on pattern analysis and machine intelligence. 2016;39(6):1137-1149.

[2] Liu W, Anguelov D, Erhan D, Szegedy C, Reed S, Fu C-Y, et al. Ssd: Single shot multibox detector. 14th European Conference on Computer Vision; 2016.

[3] Xu S, Wang X, Lv W, Chang Q, Cui C, Deng K, et al. PP-YOLOE: An evolved version of YOLO. arXiv preprint arXiv:2203.16250. 2022.

[4] Farhadi A, Redmon J. Yolov3: An incremental improvement. arXiv preprint arXiv:180402767. 2018.

[5] Bochkovskiy A, Wang C-Y, Liao H-YM. Yolov4: Optimal speed and accuracy of object detection. arXiv preprint arXiv:200410934. 2020.

[6] YOLOv5. 2023. Available: https://github.com/ultralytics/YOLOv5

[7] Li C, Li L, Jiang H, Weng K, Geng Y, Li L, et al. YOLOv6: A single-stage object detection framework for industrial applications. arXiv preprint arXiv:2209029 76. 2022.

[8] Wang C-Y, Bochkovskiy A, Liao H-YM. YOLOv7: Trainable bag-of-freebies sets new state-of-the-art for real-time object detectors. Proceedings of the IEEE/CVF conference on computer vision and pattern recognition; 2023.

[9] Zhu J, Zhang J, Wang Y, Ge Y, Zhang Z, Zhang S. Fire Detection in Ship Engine Rooms Based on Deep Learning. Sensors. 2023;23(14).

[10] YOLOv8. 2023. Available: https://github.com/ultralytics/ultralytics

[11] Cheng T, Sone L, Ge Y, Liu W, Wang X, Shan Y, et al. YOLO-World: Real-Time OpenVocabulary Object Detection. IEEE/CVF Conference on Computer Vision and Pattern Recognition (CVPR); 2024.

[12] Chen J, Kao Sh, He H, Zhuo W, Wen S, Lee CH, et al. Run, don't walk: chasing higher FLOPS for faster neural networks. Proceedings of the IEEE/CVF confer- ence on computer vision and pattern recognition; 2023.

[13] Wang C-Y, Yeh I-H, Liao H-YM. Yolov9: Learning what you want to learn using programmable gradient information. arXiv preprint arXiv:240213616. 2024.

[14] Wang A, Chen H, Liu L, Chen K, Lin Z, Han J, et al. YOLOv10: Real-Time End-to-End Object Detection. arXiv preprint arXiv:240514458. 2024.

[15] YOLOv11. 2024. Available: https://github.com/ultralytics/ultralytics

[16] Qin D, Leichner C, Delakis M, Fornoni M, Luo S, Yang F, et al. MobileNetV4: Universal Models for the Mobile Ecosystem. arXiv preprint arXiv:2404.10518. 2024.

[17] Zhang J, Li X, Li J, Liu L, Xue Z, Zhang B, et al. Rethinking Mobile Block for Efficient Attention-based Models. arXiv preprint arXiv:2301.01146. 2023.

3. Method Selection Rationale

To optimize performance for real-time fire detection on edge devices, FCMI-YOLO incorporates three key innovations that effectively balance accuracy, computational efficiency, and inference speed:

FasterNext: The FasterNext architecture, particularly its Partial Convolution (PConv), was selected due to its efficient memory access and reduced computational overhead. This makes it ideal for deployment on resource-constrained edge devices. PConv reduces the number of computations by processing only a subset of channels, significantly lowering FLOPs while maintaining feature extraction capability. Additionally, Pointwise Convolution (PWConv) is incorporated to refine feature representations after PConv operations, ensuring comprehensive feature aggregation. To further enhance non-linear expressiveness, FasterNext

employs the SiLU activation function instead of ReLU. Unlike ReLU, which suppresses negative values entirely and suffers from gradient dead zones, SiLU provides a smooth transition for negative inputs, improving gradient flow and feature learning. This property is particularly beneficial for fire detection tasks, where fire patterns exhibit complex variations.

Improvements in the Neck Network: To enhance the detection performance of YOLOv5s, particularly for small fire targets in complex environments, improvements have been made to the neck network by integrating the Cross-Scale Feature Fusion Module (CCFM) with the Mixed Local Channel Attention (MLCA) mechanism. CCFM addresses the limitations of the conventional PAN structure by employing lightweight convolutions to fuse multi-scale features. This approach not only preserves high-resolution spatial details, which are often lost due to downsampling but also facilitates cross-layer feature reuse to improve the representation of small targets even in scenarios with partial occlusions or distant fire sources. Complementing this, the MLCA module enhances detection accuracy by jointly modeling local spatial and global channel features with minimal computational overhead. By focusing on critical fire regions while effectively suppressing background noise, MLCA contributes to improved precision and reliability, particularly in cluttered or complex scenes. Together, these modules optimize the neck network to achieve superior accuracy and recall without a significant increase in computational cost, making the model well-suited for real-time fire detection on resource-constrained edge devices.

Inner-DIoU Loss Function: The Inner-DIoU loss function was selected to address the scale sensitivity in fire detection, which is a critical challenge when dealing with irregularly shaped fire sources. Unlike traditional loss functions such as CIoU, Inner-DIoU incorporates auxiliary bounding boxes with dynamically adjusted aspect ratios. This ensures better regression accuracy for both high- and low-IoU samples, enhancing the model's ability to detect small and irregular fire targets. This innovation improves the model's sensitivity to scale variations, which is particularly crucial for fire detection in diverse environments, where fire targets may vary significantly in size and shape.

These innovations were specifically chosen to enhance FCMI-YOLO's ability to perform real-time fire detection on edge devices, achieving a balance between detection performance and inference speed. The combination of FasterNext, CCFM, MLCA, and Inner-DIoU ensures that FCMI-YOLO can effectively handle complex fire detection tasks, especially in environments with small, irregularly shaped, or distant fire sources, while maintaining high inference speed and low resource consumption.

Comment 5: *The necessity & novelty of the manuscript should be presented and stressed in the Introduction section.*

Response: Thank you for your valuable suggestion. In response, we have revised the Introduction section to more clearly emphasize the necessity and novelty of our work. To highlight the necessity, we strengthened the rationale by analyzing the limitations of several recently published approaches. For example, Cao et al. [1] and Wang et al. [2] enhanced detection accuracy but introduced heavier models, making them unsuitable for real-time edge deployment. On the other hand, He et al. [3] attempted to reduce complexity but at the cost of performance robustness. These comparisons allow us to better frame the challenge: existing fire detection algorithms either sacrifice accuracy for speed or vice versa, and few achieve

both on embedded systems. To address this, we propose FCMI-YOLO, a real-time algorithm that strikes a better balance between detection precision and computational efficiency through architectural improvements and loss function optimization.

To demonstrate the novelty of our work, we explicitly state that FCMI-YOLO, an enhancement of YOLOv5s, not only improves fire detection accuracy but also optimizes inference speed, making it highly suitable for deployment on edge devices. In comparison to the aforementioned studies, our approach achieves a superior balance between accuracy and inference speed, featuring a lightweight design and efficient deployment. We highlight that FCMI-YOLO is capable of real-time detection, which is essential for practical fire detection applications in resource-constrained environments. The contribution lies in several key aspects: (1) A lightweight FasterNext module was designed to replace the C3 module in the backbone, reducing parameters and computational load while improving feature extraction in complex environments. (2) The neck network was enhanced by integrating the Cross-Scale Feature Fusion Module (CCFM) and the Mixed Local Channel Attention (MLCA) mechanism, improving small-target detection through effective multi-scale fusion and attention-based key feature enhancement. (3) To improve localization robustness, we introduce the Inner-DIoU loss function, which incorporates auxiliary bounding boxes and enhances scale awareness in bounding box regression. (4) We constructed a dedicated fire dataset to support training and evaluation, ensuring that our method performs well across various real-world fire scenarios. (5) Finally, FCMI-YOLO was successfully deployed on the Orange Pi 5 Plus edge device using an asynchronous multithreading strategy to improve NPU utilization, boosting FPS from 5.5 to 23.4 and achieving practical real-time performance.

These changes ensure that the necessity and novelty of our study are clearly presented, addressing the reviewer's concern. Please refer to the updated Introduction section for further details.

[1] Cao X, Su Y, Geng X, Wang Y. YOLO-SF: YOLO for Fire Segmentation Detection. Ieee Access. 2023;11:111079-111092.

[2] Wang S, Wu M, Wei X, Song X, Wang Q, Jiang Y, et al. An advanced multi-source data fusion method utilizing deep learning techniques for fire detection. Engineering Applications of Artificial Intelligence. 2025;142.

[3] He H, Zhang Z, Jia Q, Huang L, Cheng Y, Chen B. Wildfire detection for transmission line based on improved lightweight YOLO. Energy Reports. 2023;9:512-20.

Comment 6: *The application/theory/method/study reported is not in sufficient detail to allow for its replicability and/or reproducibility. Therefore, it is suggested to make it clear to show all steps to build the model.*

Response: Thank you for your valuable comment. In response, we have significantly expanded the details in our manuscript to ensure that every step required to build and deploy the model is clearly documented, thereby enhancing its replicability and reproducibility. Specifically, in Section 3.2, we provide a comprehensive description of the newly designed FasterNext network, including its underlying principles, its multi-branch architecture, and the roles of PConv, PWConv, and S-FasterNet in reducing computational complexity while preserving feature extraction capability. Additionally, in Section 3.3, we detail the

implementation of the Cross-Scale Feature Fusion Module (CCFM) and Mixed Local Channel Attention (MLCA) mechanism in the neck network, explaining how these components enhance multi-scale feature fusion and improve the detection of small fire targets. In Section 3.4, we provide a thorough explanation of the improved loss function, including complete mathematical formulations and a schematic of the Inner-IoU mechanism. Furthermore, the experimental sections (Sections 4.1 – 4.4) have been revised to include detailed descriptions. These revisions collectively ensure that all aspects of the model building and deployment process are clearly presented, thereby facilitating the full reproducibility of our study.

Comment 7 : *The problem statement and gap study are not clear.*

Response: Thank you for your valuable feedback. In response, we have thoroughly revised the Abstract, Introduction, and Related Work sections to clearly articulate both the problem statement and the gap study.

In the Abstract, we explicitly highlight the fundamental challenge in current fire detection research, the difficulty of achieving both high detection accuracy and real-time inference under limited computational resources. As stated:

"The rapid development of Internet of Things (IoT) technology and deep learning has propelled the deployment of vision-based fire detection algorithms on edge devices, significantly exacerbating the trade-off between accuracy and inference speed under hardware resource constraints."

This statement concisely reflects the technical bottleneck that motivates our work.

In the Introduction, we further elaborate on this challenge by analyzing the limitations of several representative researches. For example, methods proposed by Cao et al. [1] and Wang et al. [2] focus on improving detection accuracy through advanced module integration but result in increased model complexity, rendering them impractical for real-time edge deployment. Conversely, the lightweight method of He et al. [3] sacrifices detection robustness in favor of lightweight network, demonstrating the difficulty of achieving both objectives simultaneously. These researches collectively illustrate the current dilemma: most existing algorithms prioritize either speed or accuracy, with few capable of achieving an effective balance under resource-constrained conditions.

To reinforce this problem statement and gap study, we expanded the Related Work section with a structured review of YOLO-based fire detection algorithms. While recent studies using deformable attention mechanisms [4], ensemble learning [5], and multibranch fusion [6] offer accuracy improvements, they often do so at the expense of model efficiency and deployability. Even lightweight designs such as those by Wang et al. [8] and Huang et al. [9], although suitable for edge deployment, still face challenges in detecting long-range, small-scale, or partially occluded fire targets in complex scenes.

These findings highlight a clear gap in the literature: there is a lack of fire detection algorithms that simultaneously deliver high detection accuracy, low model complexity, and real-time inference speed on resource-constrained edge devices. To bridge this gap, our study proposes the FCMI-YOLO algorithm, which introduces a lightweight backbone (FasterNext), an optimized neck network (CCFM + MLCA), and a scale-aware loss function (Inner-DIoU) to achieve a better balance between detection performance and efficiency. Through

comprehensive experiments and deployment on real edge devices, we demonstrate that FCMI-YOLO effectively fills this research gap and offers a practical solution for real-time fire detection in complex scenarios.

Please refer to the updated Abstract, Introduction, and Related Work sections for further details.

Comment 8: The method is not clear. Therefore, it must be shown and clarified to show all steps.

Response: Thank you for your valuable suggestion. In response, the description of the proposed method has been substantially revised and expanded in Sections 3.1 – 3.4 to clearly present all the steps involved. Specifically, as shown in Figure 1, the modified parts of the network structure in Section 3.1 have been highlighted in red for clarity.

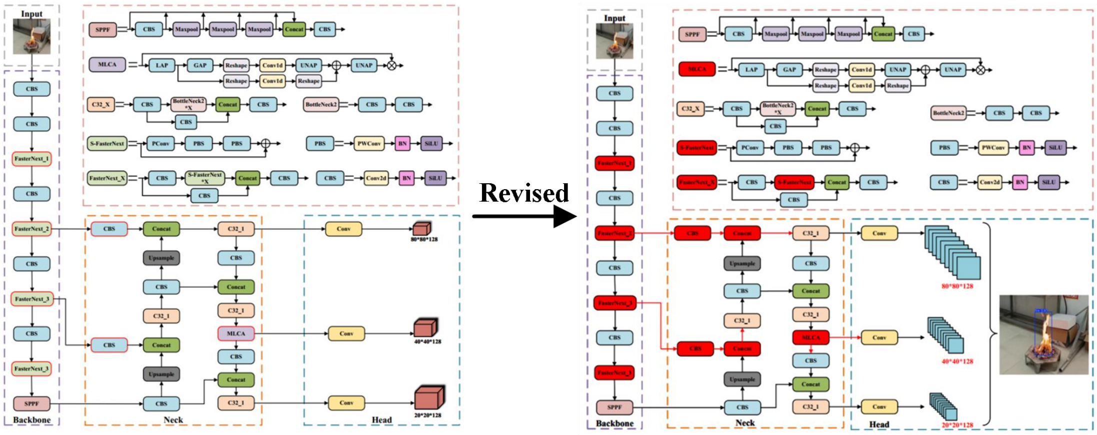

Figure 1. Comparison of the original and revised network architectures with highlighted in red.

In Section 3.2, the newly proposed FasterNext network is described in detail, including the design rationale, module structure, and key components such as PConv, PWConv, and S-FasterNet. Furthermore, supporting figures and tables have been added to illustrate the structural design, working principles, and performance comparisons with the original C3 module. This helps clarify the motivation, implementation, and performance advantages of FasterNext. In terms of theoretical analysis, we conducted an in-depth comparative study of activation functions. By introducing Equations (1) and (2), we systematically illustrated the advantages of the SiLU activation function over ReLU. These supplementary explanations help provide a more comprehensive understanding of the design motivation, implementation details, and performance benefits of the FasterNext network.

$$\mathrm{Re}LU(x) = \max(0, x) \tag{1}$$

$$SiLU(x) = \frac{x}{1+e^{-x}} \tag{2}$$

In Section 3.3.1, the introduction of the Cross-Scale Feature Fusion Module (CCFM) is explained with a focus on how it alleviates the computational burden of the PAN structure in the original YOLOv5s neck network. The process of multi-scale feature interaction, lightweight convolution design, and adaptive weight-based fusion are explicitly described to

highlight the step-by-step improvements in feature fusion for small-object fire detection tasks.

In Section 3.4, to clearly explain the rationale behind replacing the original CIoU loss function with a more suitable alternative, we introduced Equations (3)-(5). These equations provide a detailed analysis of the mathematical formulation, components, and limitations of CIoU, thereby demonstrating the necessity of improving the loss function.

$$IoU = \frac{inter}{union} \tag{3}$$

$$\nu = \frac{4}{\pi^2}\left(\arctan\frac{w^{gt}}{h^{gt}} - \arctan\frac{w}{h}\right)^2 \tag{4}$$

$$\alpha = \frac{\nu}{(1 - IoU) + \nu} \tag{5}$$

Through these revisions, the overall methodology has been systematically organized and clarified to ensure that all steps are explicitly and logically presented, thereby enhancing the reproducibility and understanding of the proposed approach.

Comment 9: *The interpretation of results and study conclusions are not supported by providing the reasons behind why they show that. Therefore, it is recommended to deepen the discussion.*

Response: Thank you for your valuable suggestion. In response, the discussion has been significantly enriched in several experimental sections of the revised manuscript.

Firstly, in Section 4.3.3 (FasterNext Comparison Experiment), a detailed rationale is provided for replacing the original C3 module in YOLOv5s with the proposed FasterNext module. Specifically, the Bottleneck structure in C3 is susceptible to feature information loss in complex fire scenarios and introduces high computational overhead, which hinders deployment on edge devices. Drawing inspiration from the lightweight design principles of FasterNet, FasterNext adopts a structure with parallel CBS and S-FasterNet modules. The introduction of partial convolution (PConv) significantly reduces FLOPs, while the use of the SiLU activation function over ReLU enhances the model’ s non-linear representation ability, particularly in complex and dynamic fire patterns.

Secondly, in Section 4.3.4 (Loss Function Comparison Experiment), comprehensive explanations are given to support the advantages of the proposed Inner-DIoU loss function. Inner-DIoU achieves the highest mAP@0.5 (88.0%) and recall (84.4%) among all compared variants. This improvement is attributed to its dynamic scaling mechanism, which adjusts the auxiliary bounding box in accordance with the actual size of the fire target. This capability enables better localization and robustness to target scale variability, which is essential in fire detection tasks due to the irregular, rapidly changing shapes and sizes of flames. Furthermore, by conducting experiments with different scaling ratios, it is demonstrated that a ratio of 0.8 yields optimal performance, supporting the design rationale of Inner-DIoU and offering insights into its effect on model learning dynamics.

Lastly, in the ablation experiments (Section 4.3.5), the individual and combined contributions of the proposed modules (FasterNext, CCFM, MLCA, and Inner-DIoU) are systematically evaluated. The analysis highlights how each component contributes to overall performance improvements. This holistic integration is crucial for achieving real-time, high-precision fire detection on edge devices.

These expanded discussions provide a more comprehensive interpretation of the experimental results, illustrating the theoretical and empirical justifications for the proposed method's superior performance.

Comment 10: *It is recommended to emphasize the strengths of the study clearly.*

Response: Thank you very much for your valuable comment. To better highlight the strengths of our proposed method, we have revised the manuscript accordingly.

First, in the Introduction section, we refined the description of related studies by incorporating a comparison of several representative and recently published fire detection algorithms. Instead of detailing individual methodologies, we summarized the typical strategies employed in existing work, such as attention mechanisms, feature fusion modules, and model compression techniques， and pointed out their common limitations. These include increased model complexity, higher computational overhead, and reduced accuracy or real-time performance when deployed on edge devices. This contextual comparison helps to underscore the balanced performance of our proposed FCMI-YOLO, which achieves both high accuracy and low resource consumption.

Second, to further support the effectiveness of FCMI-YOLO, we enhanced Section 4.3.7 by adding six new comparative experiments under various fire detection scenarios. As shown in Table 1, these experiments evaluate multiple metrics, including Precision, Recall, mAP@0.5, Parameters, and FPS. The results demonstrate that FCMI-YOLO consistently achieves superior detection performance with lower computational cost and fewer parameters, making it particularly suitable for real-time applications on resource-constrained edge devices.

In addition, the Related Work section has been expanded with a more detailed analysis of fire detection methods. While these works have made notable progress through advanced attention mechanisms, ensemble learning, and multi-branch fusion strategies, many of them still suffer from increased computational burden or performance degradation in complex environments. These comparisons further highlight the advantages of our method in balancing efficiency and accuracy.

These revisions aim to more clearly convey the innovation and practical value of our algorithm and to address the concerns regarding the clarity of our contribution.

Table 1. Performance comparison of mainstream algorithms.

Model	Parameters(M)	FLOPs(G)	Recall(%)	mAP@0.5(%)	FPS
Faster R-CNN	136.7	369.7	76.5	67.8	13.5
SSD	23.6	174.8	69.7	86.1	42.3
PP-YOLOEs	7.9	17.36	60.6	88.2	86.4
YOLOv3	62.4	65.9	78.1	82.0	28.9
YOLOv4	64.4	60.6	81.4	87.9	24.3
YOLOv5s	7.0	15.9	78.7	86.5	98.8
YOLOv6s	17.3	44.1	83.3	88.5	77.8
YOLOv7	37.2	105.1	71.3	77.6	78.3
YOLOv7-Tiny	6.0	13.0	83.9	86.4	66.1
YOLOv8s	11.1	28.6	80.7	87.7	84.0
YOLOv8s-World	13.4	32.6	82.2	89.4	36.3
YOLOv8s-FasterNet	8.6	21.8	79.6	86.1	78.4
YOLOv9s	9.9	40.6	82.9	89.0	44.2
YOLOv10s	8.1	24.8	76.7	83.1	48.1
YOLOv11s	9.4	21.5	80.3	88.3	42.5
YOLOv11s-MobileNetv4	5.2	10.5	76.3	84.1	76.0
YOLOv11s-EMO	8.5	11.5	82.0	87.9	31.5
FCMI-YOLO	4.2	11.3	84.4	88.0	91.2

Comment 11: *The limitations of the study should be stated.*

Response: Thank you for your valuable feedback. In response, we have revised the Conclusion to clearly state the limitations of our study. The updated Conclusion now acknowledges that while FCMI-YOLO achieves a significant balance between accuracy and inference speed, its detection accuracy may decrease when fire targets are distant or in harsh environments (such as strong winds, intense lighting, or reflective surfaces). Moreover, although the model has been optimized in terms of parameter efficiency and computational cost, there is still room for further improvements in inference speed, especially for real-time applications on certain hardware. Finally, we recognize that the performance of edge devices can vary depending on their specific hardware configurations, which presents a challenge for universal deployment. These revisions ensure that the study's limitations are explicitly communicated while also highlighting potential directions for future research.

Comment 12: *The manuscript structure, flow, or writing needs some improvements.*

Response: We thank the reviewer for pointing out the issues related to the structure, logical flow, and writing quality of the manuscript. In response, we have carefully revised the manuscript to improve its organization, coherence, and overall clarity. Specifically, we rewrote the Abstract to provide a more comprehensive summary of the motivation, method, experimental results, and limitations, avoiding unnecessary repetition. The Introduction has been reorganized to present a more logical progression from traditional fire detection methods to the necessity of deploying lightweight deep learning algorithms on edge devices. In the Related Work section, we incorporated recent and relevant literature to better position our research within the existing body of work.

We also enhanced the methodological descriptions by providing clearer explanations of key modules such as FasterNext, CCFM, MLCA, and the proposed Inner-DIoU loss, allowing readers to better understand the novelty and rationale of our approach. In addition, we included new comparative experiments to further support the effectiveness of our proposed algorithm. Finally, the Conclusion has been revised to not only summarize the main findings but also acknowledge the limitations of the current work and suggest future research directions. We believe that these improvements have significantly strengthened the quality and readability of the manuscript, and we sincerely appreciate the reviewer’s helpful

comments.

Comment 13: *The manuscript is benefit from language editing. The English of the paper is readable; however, I would suggest the authors to have it checked preferably by a native English-speaking person to avoid any mistakes.*

Response: Thank you for your suggestion. We have carefully reviewed and revised the manuscript to improve language clarity and correctness, and we believe the overall readability of the paper has been enhanced. In the revised version, all language-related modifications have been clearly highlighted to facilitate review. We appreciate your feedback and will continue to work on further language refinements in future revisions.

Comment 14: *I noticed that the conclusion section tends to repeat the Abstract and results. The conclusion paragraph should be short, impactful, and direct the reader to this research's next steps and opportunities.*

Response: Thank you for your valuable feedback. In response, we have revised the Conclusion to make it more concise and impactful, avoiding unnecessary repetition of the Abstract and Results sections while clearly directing the reader toward future research directions. The revised Conclusion now succinctly summarizes our key contributions, the introduction of the lightweight FasterNext module, the integration of the CCFM and MLCA mechanisms, and the proposal of the Inner-DIoU loss function demonstrating how these innovations enable FCMI-YOLO to achieve a balanced trade-off between accuracy and inference speed on both PCs and edge devices. Additionally, we have incorporated a brief discussion of the method's limitations, specifically noting that detection accuracy may decrease in adverse conditions (e.g., long-distance fires, harsh environments) and that inference speed improvements remain possible, particularly given the variability in edge device hardware. Finally, the revised Conclusion outlines future research directions, emphasizing the need to enhance the generalization ability of FCMI-YOLO and further explore lightweight strategies to improve real-time performance. We believe these revisions effectively address the comment and provide a clear and forward-looking conclusion. Please refer to the updated Conclusion in the revised manuscript for further details.

Comment 15: *It will be nice to add some new references to show that your study is updated, such as:*

Zhou, Zhanxin, and Ruibo Wu. "Stock Price Prediction Model Based on Convolutional Neural Networks." Journal of Industrial Engineering and Applied Science 2.4 (2024): 1-7;

Alakbari, Fahd Saeed, et al. "Prediction of critical total drawdown in sand production from gas wells: Machine learning approach." The Canadian Journal of Chemical Engineering 101.5 (2023): 2493-2509.;

Alakbari, Fahd Saeed, et al. "Deep learning approach for robust prediction of reservoir bubble point pressure." ACS omega 6.33 (2021): 21499-21513.;

Alakbari, Fahd Saeed, et al. "A gated recurrent unit model to predict Poisson's ratio using deep learning." Journal of Rock Mechanics and Geotechnical Engineering 16.1 (2024): 123-135;

Wu, Ruibo, Tao Zhang, and Feng Xu. "Cross-Market Arbitrage Strategies Based on Deep

Learning." Academic Journal of Sociology and Management 2.4 (2024): 20-26.

Response: We sincerely thank the reviewer for the constructive suggestion. It is indeed a valuable point that incorporating the latest relevant literature can enhance the timeliness and completeness of our study. These references are very helpful for us to better understand the recent advancements in deep learning-based prediction and modeling. In the revised manuscript, we have added and cited the following references:

[1] Zhou, Zhanxin, and Ruibo Wu. "Stock Price Prediction Model Based on Convolutional Neural Networks." Journal of Industrial Engineering and Applied Science 2.4 (2024): 1-7;

[2] Wu, Ruibo, Tao Zhang, and Feng Xu. "Cross-Market Arbitrage Strategies Based on Deep Learning." Academic Journal of Sociology and Management 2.4 (2024): 20-26.

[3] Alakbari FS, Mohyaldinn ME, Ayoub MA, Muhsan AS. Deep learning approach for robust prediction of reservoir bubble point pressure. ACS omega. 2021;6(33):21499-513.

[4] Alakbari FS, Mohyaldinn ME, Ayoub MA, Muhsan AS, Abdulkadir SJ, Hussein IA, et al. Prediction of critical total drawdown in sand production from gas wells: Machine learning approach. The Canadian Journal of Chemical Engineering. 2023;101(5):2493-509.

[5] Alakbari FS, Mohyaldinn ME, Ayoub MA, Hussein IA, Muhsan AS, Ridha S, et al. A gated recurrent unit model to predict Poisson's ratio using deep learning. Journal of Rock Mechanics and Geotechnical Engineering. 2024;16(1):123-35.

These citations have been incorporated into the revised manuscript to support the discussion on the broad applicability and generalizability of deep learning models, especially in industrial IoT. Specifically, in Section Related Work, we added the following sentence:

"Convolutional neural network (CNN)-based methods can automatically extract multi-scale abstract features from large-scale data, and their hierarchical feature representation effectively mitigates sensitivity to environmental interference [1, 2]. "

"Consequently, they have been widely ap- plied in various fields, including autonomous driving, smart agriculture, security survei- llance, and industrial IoT [3,4,5]. "

We appreciate the reviewer's insightful recommendations, which have helped improve the quality and academic rigor of our manuscript.

Response to Associate Editor's Comments

Comment 1: *Please ensure that your manuscript meets PLOS ONE's style requirements, including those for file naming.*

Response: Thank you for your comments. I have reviewed the PLOS ONE style requirements and have formatted the manuscript according to the provided templates. The manuscript now adheres to the guidelines specified in the links you provided.

Comment 2: *Please note that PLOS ONE has specific guidelines on code sharing for submissions in which author-generated code underpins the findings in the manuscript. In these cases, we expect all author-generated code to be made available without restrictions upon publication of the work. Please review our guidelines at https://journals.plos.org/plosone/s/materials-and-software-sharing#loc-sharing-code and*

ensure that your code is shared in a way that follows best practice and facilitates reproducibility and reuse.

Response: I acknowledge PLOS ONE's guidelines on code sharing. I confirm that all author-generated code underlying the findings in this manuscript will be made publicly available upon publication, following best practices to ensure reproducibility and reuse. The core code will be provided in a public repository, with the relevant details included at the end of the abstract. The core code and dataset required for the experiment are saved in this article at https://github.com/JunJieLu20230823/code.git.

Comment 3: *In the online submission form, you indicated that the data cannot be shared publicly because they involve subsequent applications for patents, software copyright, and the publication of project deliverables. The data underlying the results presented in this study are available upon request from the corresponding author. All PLOS journals now require all data underlying the findings described in their manuscript to be freely available to other researchers, either 1. In a public repository, 2. Within the manuscript itself, or 3. Uploaded as supplementary information. This policy applies to all data except where public deposition would breach compliance with the protocol approved by your research ethics board. If your data cannot be made publicly available for ethical or legal reasons (e.g., public availability would compromise patient privacy), please explain your reasons on resubmission and your exemption request will be escalated for approval.*

Response: Regarding data availability, I agree to make the data underlying the findings in this study publicly available, in accordance with PLOS ONE's policy. The data will be uploaded to a public repository, ensuring full transparency and accessibility for other researchers. The core code and dataset required for the experiment are saved in this article at https://github.com/JunJieLu20230823/code.git.
