# Supplementary material for: FCMI-YOLO: An efficient deep learning-based algorithm for real-time fire detection on edge devices
Source: PLoS One. 2025 Aug 7;20(8):e0329555. doi: 10.1371/journal.pone.0329555 (PMC12331093; doi:10.1371/journal.pone.0329555)
Supplement: Attachment — Submitted filename: Response_to_Reviewers_auresp_2.pdf [file pone.0329555.s002.pdf]

Response to Reviewers' Comments

<u>Manuscript ID</u>: PONE-D-25-01361R1

<u>Title</u>: FCMI-YOLO: An Efficient Deep Learning-Based Algorithm for Real-Time Fire Detection on Edge Devices

<u>Authors</u>: Junjie Lu, Yuchen Zheng, Liwei Guan, Bing Lin, Wenzao Shi, Junyan Zhang and Yunping Wu

We would like to thank the editor for handling the review process of our manuscript. We are very grateful to the reviewers for their thorough review and constructive comments. We have carefully addressed all the review comments and improved the quality of this paper accordingly. Please find below our response to review comments one by one.

In the Revised Manuscript with Track Changes, all modifications are highlighted in yellow for reference.

Response to Reviewer 1's Comments

Comment 1: The authors should emphasize the merits of the developed method.

Response: We thank the reviewer for this valuable suggestion. In the revised manuscript, we have further emphasized the advantages of the proposed FCMI-YOLO algorithm from multiple aspects:

In the last paragraph of the "Related Work" section, we explicitly highlight the unique positioning of FCMI-YOLO compared to existing accuracy and lightweight algorithms, stating that it effectively achieves a trade-off between detection precision and inference speed, which is essential for edge deployment.

In Section 4.3.7 (Comparison of Mainstream Algorithms), we provide detailed comparisons with 17 mainstream algorithms in terms of precision, recall, inference speed, parameters, and FLOPs. These comparisons demonstrate that FCMI-YOLO outperforms most algorithms with only 4.2M parameters and 11.3G FLOPs, achieving 88.0% mAP@0.5 and 91.2 FPS. Additionally, we present visual detection comparisons under challenging conditions (e.g., occlusion, long-range), where our method shows superior robustness and minimal missed detections.

In Section 4.4.3 (Deployment Results), we detail outdoor experiments on the OrangePi 5 Plus, where FCMI-YOLO achieves higher detection accuracy than YOLOv5s at both 30m and 75m distances while maintaining real-time performance (23.4 FPS). These results clearly validate the algorithm's practical effectiveness and suitability for deployment on resource-constrained platforms.

To further underscore these merits, we have added a summarizing paragraph at the end of Section 4.3.7 that articulates FCMI-YOLO's balance between lightweight design, high accuracy, and real-time inference, especially under edge computing constraints. This helps to clearly position the proposed method in contrast to other mainstream algorithms.

Comment 2: In the section simulation results, the proposed approach should be compare with other relevant algorithms, the authors are asked to further provide comparisons to better show the advantages of their approach.

Response: We appreciate the reviewer's insightful suggestion regarding the need to further highlight the advantages of the proposed algorithm through comparative analysis.

In response, we have significantly enhanced the simulation results in two key parts to address this issue:

1. Section 4.3.7 (Comparison of Mainstream Algorithms):

We conducted a detailed comparison between FCMI-YOLO and 17 mainstream algorithms, including Faster R-CNN, SSD, YOLOv3 to v11s variants, and several lightweight or enhanced versions such as YOLOv8s-World and YOLOv8s-FasterNet. Metrics such as model parameters, FLOPs, mAP@0.5, recall, and FPS were evaluated. The results show that FCMI-YOLO achieves the highest recall (84.4%) and maintains competitive precision (88.0%) while using significantly fewer parameters (4.2M) and computational cost (11.3 GFLOPs). As shown in Figure 13 of the revised manuscript, FCMI-YOLO exhibits more robust detection than other advanced YOLO variants under challenging fire scenarios.

2. Section 4.4.3 (Deployment Results):

To validate the algorithm's effectiveness on edge devices, we performed deployment experiments on the OrangePi 5 Plus. We compared FCMI-YOLO against YOLOv5s at distances of 30 m and 75 m from fire sources. FCMI-YOLO achieved higher detection precision (88% and 70%) than YOLOv5s (72% and 59%), with only a minor decrease in FPS. These findings demonstrate the superior robustness and stability of our algorithm under resource-constrained devices.

Through these enhanced comparative experiments and visual analyses, we believe the advantages of FCMI-YOLO in terms of detection accuracy, real-time performance, and hardware adaptability are now more clearly demonstrated in the revised manuscript.

Comment 3: The literature review is not good enough.

Response: Thank you for your constructive feedback. In response to your suggestion, we have thoroughly revised the Related Work section to enhance its clarity, structure, and completeness.

The revised section now provides a more comprehensive overview of existing fire detection methods, beginning with a comparison between traditional handcrafted feature-based methods and deep learning-based methods. We further classify CNN-based detection algorithms into two-stage and one-stage algorithms and discuss their respective advantages and limitations in real-time fire detection applications.

In addition, we have reorganized and expanded the review of recent YOLO-based fire detection models. These works are now grouped according to their core contributions, including accuracy-oriented enhancements (for example, DG-YOLO, YOLOX-CBAM, FSDF, and YOLOv5 combined with EfficientDet) and lightweight optimization for edge deployment (such as Light-YOLOv4 and YOLO-ULNet).

Finally, we summarize the common trade-offs observed in current research, particularly the difficulty of balancing detection precision and computational efficiency, which motivates the development of the proposed FCMI-YOLO algorithm aimed at addressing these challenges on edge devices.

Comment 4: Some minor grammatical errors should be eliminated. The authors are kindly

asked to double check the manuscript.

Response: We sincerely thank the reviewer for pointing this out. In response, we have carefully rechecked and polished the manuscript to improve its grammatical accuracy and language clarity. Special attention has been paid to sentence structure, verb usage, punctuation, and overall readability to enhance the quality and professionalism of the writing.

Comment 5: The abstract could be more concise and the point.

Response: Thank you for your valuable suggestion. In response, we have revised the abstract to make it more concise and focused on the core contributions of our work. Redundant descriptions have been removed, and the presentation of technical innovations and experimental results has been streamlined to enhance clarity and readability. The updated abstract clearly highlights the key components of our proposed method, namely FasterNext, CCFM, MLCA, and Inner-DIoU, and presents the performance improvements in a direct and succinct manner.

Original Abstract:

The rapid development of Internet of Things (IoT) technology and deep learning has propelled the deployment of vision-based fire detection algorithms on edge devices, significantly exacerbating the trade-off between accuracy and inference speed under hardware resource constraints. To address this issue, this paper proposes a real-time fire detection algorithm named FCMI-YOLO. Firstly, the FasterNext model, which is based on FasterNet, is introduced to reduce the computational of model parameters and enhance detection precision through lightweight design. Secondly, the Cross-Scale Feature Fusion Module (CCFM) and the Mixed Local Channel Attention (MLCA) mechanism are incorporated into the neck network, significantly improving detection performance for small fire targets and minimizing resource consumption. Finally, the Inner-DIoU loss function is proposed to optimize bounding box regression by incorporating auxiliary boxes. Experimental results on the fire dataset we constructed demonstrate that FCMI-YOLO increases mAP@50 by 1.5%, decreases the number of parameters by 40%, and lowers GFLOPs to 28.9% of those in YOLOv5s. These results demonstrate that FCMI-YOLO enhances fire detection accuracy while significantly reducing computational demands, making it highly valuable for widespread practical applications, particularly on resource-constrained edge devices. The core code and dataset required for the experiment are saved in this article at https://github.com/JunJieLu20230823/code.git.

Revised Abstract:

The rapid development of Internet of Things (IoT) technology and deep learning has propelled the deployment of vision-based fire detection algorithms on edge devices, significantly exacerbating the trade-off between accuracy and inference speed under hardware resource constraints. To address this issue, this paper proposes FCMI-YOLO, a real-time fire detection algorithm optimized for edge devices. Firstly, the FasterNext module is proposed to reduce computational cost and enhance detection precision through lightweight design. Secondly, the Cross-Scale Feature Fusion Module (CCFM) and the Mixed Local Channel Attention (MLCA) mechanism are incorporated into the neck network to improve detection performance for small fire targets and reduce resource consumption. Finally, the Inner-DIoU loss function is proposed to optimize bounding box regression. Experimental results on a custom fire dataset demonstrate that FCMI-YOLO increases mAP@50 by 1.5%, reduces parameters by 40%, and lowers GFLOPs to 28.9% of YOLOv5s, demonstrating its practical value for real-time fire detection in edge scenarios

with limited computational resources. The core code and dataset are available at https://github.com/JunJieLu20230823/code.git.

Comment 6: I suggest the authors consider exposes the equation more clearly so to achieve a broader audience.

Response: Thank you very much for your insightful suggestion. In response, we have thoroughly revised the mathematical formulations and their corresponding explanations in the manuscript to enhance clarity and accessibility for a broader audience. Specifically, in Section 3.2 (FasterNext Network), we improved the presentation of the ReLU and SiLU activation functions by providing clearer definitions, highlighting their respective limitations, and explaining the rationale for adopting SiLU over ReLU. To aid intuitive understanding, a comparative plot is provided in Figure 4 of the revised manuscript and the accompanying text has been refined to more effectively capture the interest of readers. Additionally, in Section 3.4 (Improved Loss Function), we restructured the explanation of the CIoU, DIoU, and proposed Inner-DIoU loss functions. We clarified the roles and interactions of key parameters and emphasized their importance in fire detection tasks involving small or irregular targets. Moreover, we explicitly discussed the limitations of CIoU in such scenarios and the motivation for proposing Inner-DIoU. To further support understanding, Figure 7 in the revised manuscript presents a schematic diagram illustrating the concept of the inner bounding box used in the Inner-IoU calculation. These revisions aim to make the mathematical components of the manuscript more transparent and accessible to a wider range of readers.

Comment 7: Please add more sentences to describe the limitations of this work. The advantages and disadvantages should be discussed.

Response: Thank you for your valuable feedback. In response to your suggestion, we have revised the conclusion section to explicitly discuss the limitations of the proposed method and to present a balanced view of its advantages and disadvantages. Specifically, we have added three key limitations. First, in challenging environments such as strong wind or reflective surfaces, fire deformation, and mirror reflection of fire may interfere with feature extraction, leading to increased false positives and reduced detection accuracy. Second, although the proposed model has been optimized for lightweight deployment and achieves real-time performance on edge devices, its inference speed still requires further improvement to support more demanding real-time applications. Third, the performance of FCMI-YOLO may vary significantly across different hardware platforms, presenting a challenge for cross-platform optimization and deployment. In addition, we have clarified that future research will focus on enhancing robustness under complex environmental conditions, exploring more aggressive lightweight strategies to improve generalization and scalability.
